# Discovery and optimized extraction of the anti-osteoclastic agent epicatechin-7-O-β-D-apiofuranoside from *Ulmus macrocarpa* Hance bark

**DOI:** 10.1038/s41598-023-38208-4

**Published:** 2023-07-09

**Authors:** Chanhyeok Jeong, Yeon-Jin Cho, Yongjin Lee, Weihong Wang, Kyu-Hyung Park, Eun Roh, Chang Hyung Lee, Young-Jin Son, Jung Han Yoon Park, Heonjoong Kang, Ki Won Lee

**Affiliations:** 1 Department of Agricultural Biotechnology and Research Institute of Agriculture and Life Sciences, Seoul National University, Seoul, 08826 Korea; 2 Bio-MAX Institute, Seoul National University, Seoul, 08826 Korea; 3 Department of Pharmacy, Sunchon National University, 315 Maegok-dong, Suncheon, Jeollanam-do 57922 Korea; 4 Laboratory of Marine Drugs, School of Earth and Environmental Sciences, Seoul National University, NS-80, Seoul, 08826 Korea; 5 Research Institute of Oceanography, Seoul National University, NS-80, Seoul, 08826 Korea; 6 Interdisciplinary Graduate Program in Genetic Engineering, Seoul National University, NS-80, Seoul, 08826 Korea; 7 Advanced Institutes of Convergence Technology, Seoul National University, Suwon, 16229 Korea; 8 Institutes of Green Bio Science and Technology, Seoul National University, Pyeongchang, 25354 Korea; 9 Department of Agricultural Biotechnology and Center for Food and Bio convergence, Seoul National University, Seoul, 08826 Korea

**Keywords:** Chemical biology, Drug discovery

## Abstract

*Ulmus macrocarpa* Hance bark (*Um*Hb) has been used as a traditional herbal medicine in East Asia for bone concern diseases for a long time. To find a suitable solvent, we, in this study, compared the efficacy of *Um*Hb water extract and ethanol extract which can inhibit osteoclast differentiation. Compared with two ethanol extracts (70% and 100% respectively), hydrothermal extracts of *Um*Hb more effectively inhibited receptor activators of nuclear factor κB ligand-induced osteoclast differentiation in murine bone marrow-derived macrophages. We identified for the first time that (2R,3R)-epicatechin-7-O-β-D-apiofuranoside (E7A) is a specific active compound in *Um*Hb hydrothermal extracts through using LC/MS, HPLC, and NMR techniques. In addition, we confirmed through TRAP assay, pit assay, and PCR assay that E7A is a key compound in inhibiting osteoclast differentiation. The optimized condition to obtain E7A-rich *Um*Hb extract was 100 mL/g, 90 °C, pH 5, and 97 min. At this condition, the content of E7A was 26.05 ± 0.96 mg/g extract. Based on TRAP assay, pit assay, PCR, and western blot, the optimized extract of E7A-rich *Um*Hb demonstrated a greater inhibition of osteoclast differentiation compared to unoptimized. These results suggest that E7A would be a good candidate for the prevention and treatment of osteoporosis-related diseases.

## Introduction

Twenty percent of all bones are replaced every year through a continuous balancing between bone formation by osteoblasts and bone resorption by osteoclasts^1^. This continuous process called bone remodeling can maintain the shape, quality, and size of skeleton. Unlike the appearance of a porous mineralized structure, bone remodeling is designed to maintain the healthy state of bone tissue by repeating destruction and resorption proceedings through a continuous active metabolic system centered on the interaction between osteoclasts and osteoblasts. Osteoclasts are multinucleated cells (MNCs) derived from the differentiation of monocyte-macrophage lineages that reabsorb bones through multiple steps. When the resorption of osteoclasts proceeds faster than bone production by osteoblasts, the bone density and components of the bone itself decrease, which leads to bone diseases^2^.

The genus *Ulmus* in the family *Ulmaceae* inhabits temperate and tropical mountainous regions of the United States, Eurasia, and the Middle East, and has flourished and spread throughout much of the Northern Hemisphere. More than 40 *Ulmus* species are distributed worldwide. Representative species native to Korea include *Ulmus pumila* L, *Ulmus parvifolia* Jacq, *Ulmus davidiana Planchon*, *Ulmus davidiana var. japonica* (Rehder) *Nakai*, and *U. macrocarpa* Hance. In particular, *Ulmus macrocarpa* Hance bark (*Um*Hb) also known as Yubaekpi, Yupi and Yugeunpi is the husk of a plant consumed in East Asia in the form of tea as herbal medicine after being dried and ground. These plants are also called nose trees because they have been known to improve rhinitis since the ancient time when they were consumed as tea^3^.

Recently, crude extracts of *U. macrocarpa* Hance were studied for hyperlipidemia modulation^4^, ulcerative colitis treatment^5^, anticoccidial effects^6^, anti-hypertensive properties^7^, and attenuated H_2_O_2_ and UVB-induced skin photoaging^8^. Research on treating bone-related diseases using trees from the other genus *Ulmus* has generally focused on promoting osteoblasts' growth and proliferation or inhibiting osteoclasts' differentiations. Previous studies on another genus) showed that *U. davidiana Planch* extracts promoted osteoblast differentiation^9^, whereas flavonoids isolated from the bark of *Ulmus wallichiana* stimulated osteoblast function and inhibited osteoclast and adipocyte differentiation^10^. Quercetin-6-C-β-D-glucopyranoside from *U. wallichiana Planchon* has been reported to potently inhibit osteoclast formation and improve ovariectomy-induced bone loss in mice^11^.

In a previous study, our group found active compounds in the ethanol extract of *Um*Hb that inhibit osteoclast differentiation^12^. Since Asians drink Yubaekpi as hot tea or as herbal medicine, we, in the current study, investigated the ability of the hot water extract of *Um*Hb to inhibit osteoclast differentiation. A compound not found in ethanol extracts in previous studies was purified and identified by high-performance liquid chromatography (HPLC), mass spectrometry (MS), and nuclear magnetic resonance (NMR) techniques. In this study, for the first time, the active compound of the hot water extract of *Um*Hb was isolated and identified as (2R,3R)-epicatechin-7-O-β-D-apiofuranoside (E7A). To our knowledge, this is also the first report that E7A extracted from *Um*Hb with hot water inhibits osteoclast differentiation. In addition, we selected the *Ulmus* species from the bark which the most E7A could be extracted. Furthermore, the extraction method was optimized by response surface methodology (RSM) to obtain as much E7A as possible. Finally, we showed that the optimized *Um*Hb extract inhibited osteoclast differentiation in a dose-dependent manner.

## Result

### Structural elucidation of the active compound

**Figure 1:**
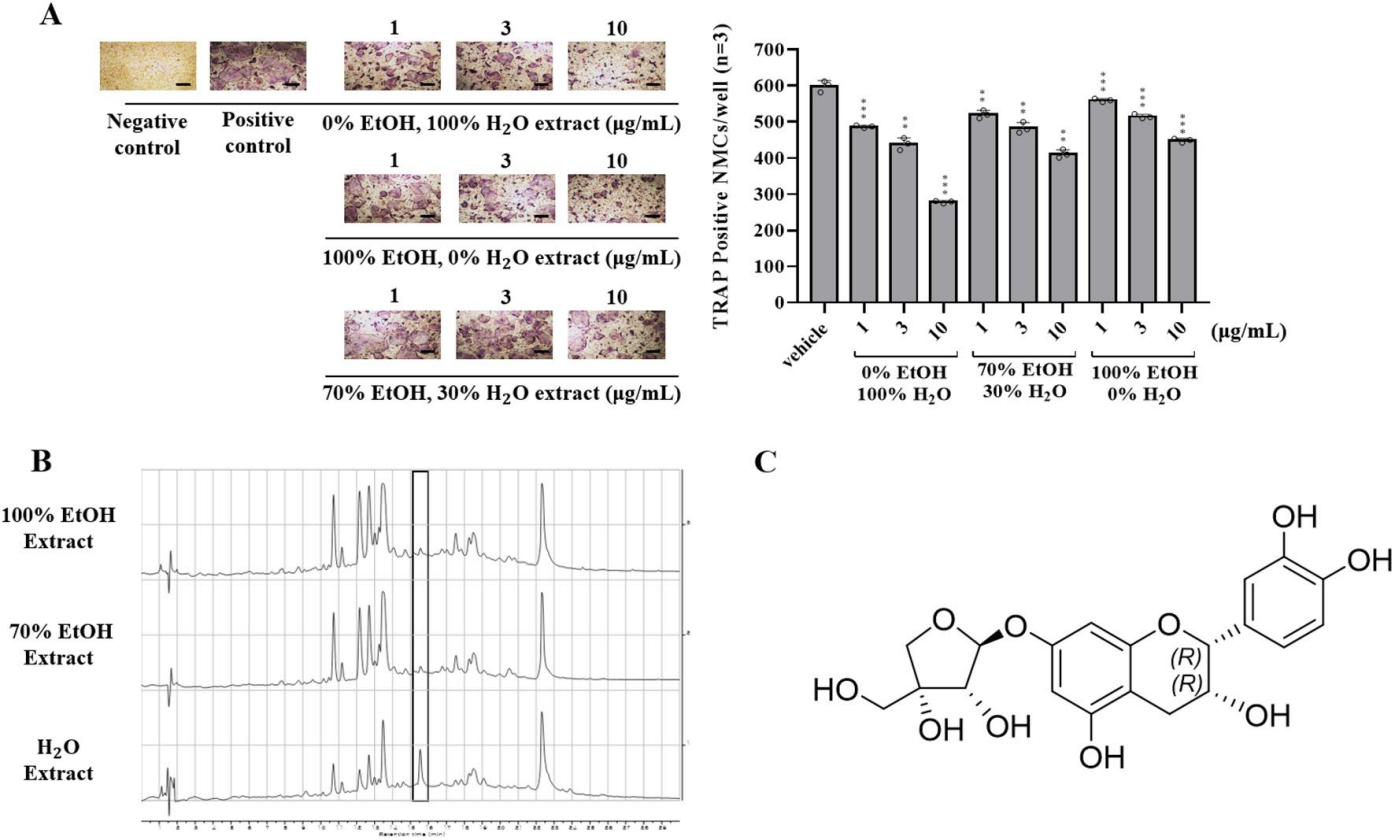
(**A**) Effects of *Um*Hb extracts (1, 3 and 10 µg/mL) on RANKL-induced osteoclast differentiation. TRAP-positive MNCs (nuclear number > 3) were counted. (**B**) Comparison of LC–MS chromatograms of *Um*Hb extracts (1 mg/mL) obtained with different solvents. A high-intensity peak was observed in the water extract at 15.53 min but not in the 70% or 100% ethanol extract. (**C**) Identified compound E7A ((2R,3R)-epicatechin-7-O-β-D-apiofuranoside). Data are mean ± SD (n = 3). *P < 0.05, **P < 0.01, ***P < 0.001 versus the vehicle (positive control) by Student’s t-test. Scale bar, 500 μm.

In this study, we first compared the ability of the hot water extract to inhibit osteoclast differentiation with that of a 70% (or 100%) ethanol extract. 300 mg of dried raw material (*Um*Hb) was added to 30 mL of distilled water, 70% ethanol, or 100% ethanol at 70 °C, followed by extraction for 120 min. As shown in Fig. 1A, the hot water extracts inhibited RANKL-induced osteoclast differentiation more efficiently than the ethanol extracts. The 100% water extract reduced the number of TRAP-positive BMCs/well by 26.72% compared to the vehicle even at a concentration as low as 3 µg/mL. Under the experimental conditions of the previous study, 100% ethanol extract showed a 14.02% reduction at 3 µg/mL, and 70% ethanol extract was reduced by 19.28% at the same dose. In addition, at the concentration of 10 µg/mL of 100% water extract, the number of TRAP-positive BMCs/well was reduced by 53.53%, showing a significantly higher inhibitory effect on osteoclast differentiation compared to other extracts at the same dose. None of the test extracts evaluated by the Cell Counting Kit-8 (CCK-8) assay showed significant cytotoxic effects on against BMCs at 1–20 μg/mL (Supplementary Fig. S1). We used LC–MS to find compounds of the three different extracts. A sharp peak (15.53 min) was found in the hydrothermal extract conspicuously, which was rarely seen in the 70% and 100% ethanol extracts (Fig. 1B). 1H NMR, 13C NMR, COSY, HSQC, and HMBC spectra were used to identify and purify the compound (Supplementary Figs. 2–5).

The isolated compound from the *Um*Hb hydrothermal extract (Fig. 1C) was composed of a flavan-3-ol aglycone and a sugar moiety and showed UV absorptions at 203 nm (maximum) and 280 nm (maximum). Its molecular formula of C_20_H_22_O_10_ was reduced by positive and negative ESI MS molecular ion peaks at m/z 423 [M + H]+ and 421 [M − H]− , respectively. The 1H NMR spectrum gave two overlapping aromatic singlets at δ 6.13 for H-6 and H-8 from a 1,2,3,5-tetrasubstituted benzene ring and three aromatic signals at δ 6.98, 6.80 and 6.76 from an ABX spin system. The proton signal of H-2 was observed as a broad singlet signal at δ 4.82 in methanol-d4 and a doublet at δ 4.72 with a small coupling constant of 3.5 Hz in DMSO-d6, which revealed a 2,3-cis relative configuration. A 2R, 3R absolute configuration was suggested by the optical rotation of − 19.6 ([α]22D). The remaining five aliphatic proton signals were observed at δ 5.49, 4.14, 4.09, 3.85, and 3.62, which suggested the presence of a D-apiofuranose. The β sugar linkage was determined based on the coupling constant (J = 2.9 Hz) of the anomeric proton of the apiofuranose moiety. The 7-O-linkage of the sugar moiety at C-7 was confirmed by the HMBC correlations from H-1'' to its neighboring carbon atoms C-6, C-7, and C-8. This compound was identified as epicatechin-7-O-β-D-apiofuranoside (E7A).

### Inhibition of osteoclast differentiation by the isolated compound

**Figure 2:**
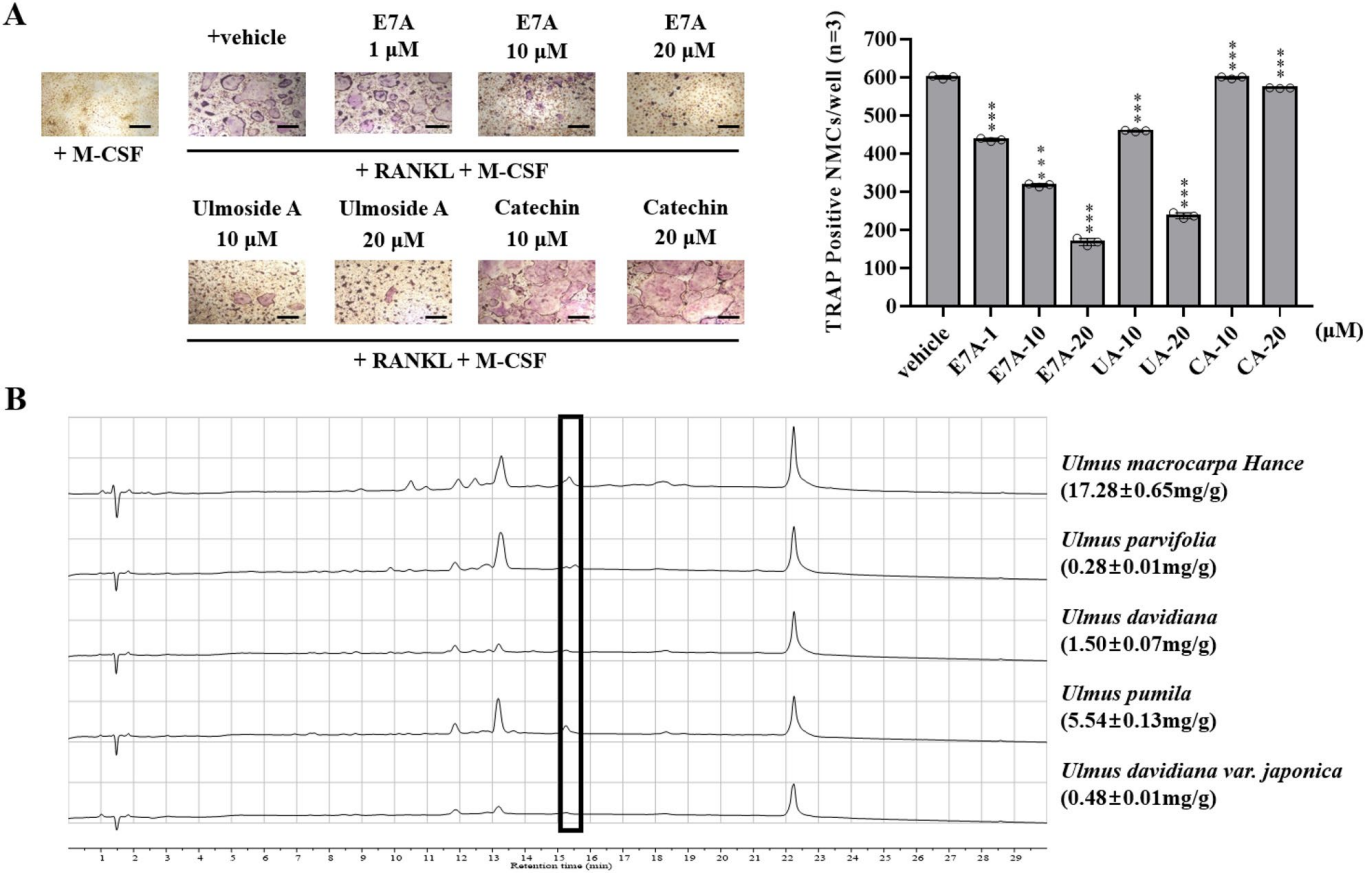
(**A**) Effects of E7A (1, 10, and 20 µM) on RANKL-induced osteoclast differentiation. Ulmoside A (UA) and catechin (CA) were used as positive controls. (**B**) Various barks of the genus *Ulmus* were extracted under the same conditions, and the E7A content in the extracts was detected at a retention time of 15.53 min through LC–MS chromatography. Data are mean ± SD (n = 3). *P < 0.05, **P < 0.01, ***P < 0.001 versus the vehicle (positive control) by Student’s t-test. Scale bar, 500 μm.

BMCs were cultured with 10 ng/mL RANKL and 30 ng/mL M-CSF for three days to generate preosteoclasts. Preosteoclasts were differentiated into TRAP-positive mature multinucleated osteoclasts (TRAP+MNCs) by incubated with M-CSF (30 ng/mL) for 30 min and RANKL (10 ng/mL) for an additional one day^12,13^. Vehicles or isolated compounds were added during this incubation step. After one day, osteoclasts were fixed and stained using a kit to calculate activity. The compound isolated from the hot water extract was identified as E7A. Next, the ability of the newly discovered compound E7A to reduce osteoclast differentiation was compared with that of ulmoside A, which is the most effective compound in the ethanol extract of *Um*Hb, and with that of catechin, which is generally regarded as the main component of bark from trees of the genus *Ulmus*. TRAP was used as a primary marker due to its high expression in activated osteoclasts. As shown in Fig. 2A, ulmoside A and E7A showed potent inhibitory activity against the RANKL-induced differentiation of BMCs into mature osteoclasts, whereas catechins had no effect. Ulmoside A and E7A inhibited RANKL-induced BMC differentiation by 23.43% and 47.13%, respectively, at 10 μM (Fig. 2A). The most potent compound, E7A, dose-dependently inhibited RANKL-induced osteoclast differentiation when tested at concentrations as low as 1 μM (Fig. 2A). IC50 data of ulmoside A and E7A was 10.3 μM and 6.6 μM as shown in Fig. 2. None of the test compounds evaluated by using the Cell Counting Kit-8 (CCK-8) assay showed significant cytotoxic effects on against BMCs at 20 μM (Supplementary Fig. S6).

### Difference in the contents of E7A in the barks of different *Ulmus* species

As described above, we determined that the glycoside flavonoid E7A from the hydrothermal extract of *Um*Hb inhibits osteoclast differentiation most effectively. As a next step, an experiment was conducted to investigate the contents of E7A in the barks of different *Ulmus* species that were mixed and sold on the market. These barks were extracted under the same conditions using pretreated dry samples. The extracted solution samples were analyzed by LC/MS. A UV (210 nm) signal peak was detected at 15.53 min in all of the extracts except for the *U. davidiana var japoni*ca Nakai extract (Fig. 2B). MS experiments were conducted on the peak at 15.53 min to improve the accuracy of the qualitative experiments (Supplementary Fig. S7). E7A, which we selected as the standard and active compound, gave peaks at m/z 423 [M + H]+ and 421 [M − H]−. The MS results showed a peak at 423.150 m/z only in the *U. pumila* and *Um*Hb extracts. Using the HPLC standard curve method, the content of E7A (mg) per extract (g) was measured to be 5.54 mg/g in the *U. pumila* extract and 17.28 mg/g in the *Um*Hb extract, showing an approximately 3.12-fold difference.

### Effect of E7A on inhibiting RANKL-induced NFATC1 expression

**Figure 3:**
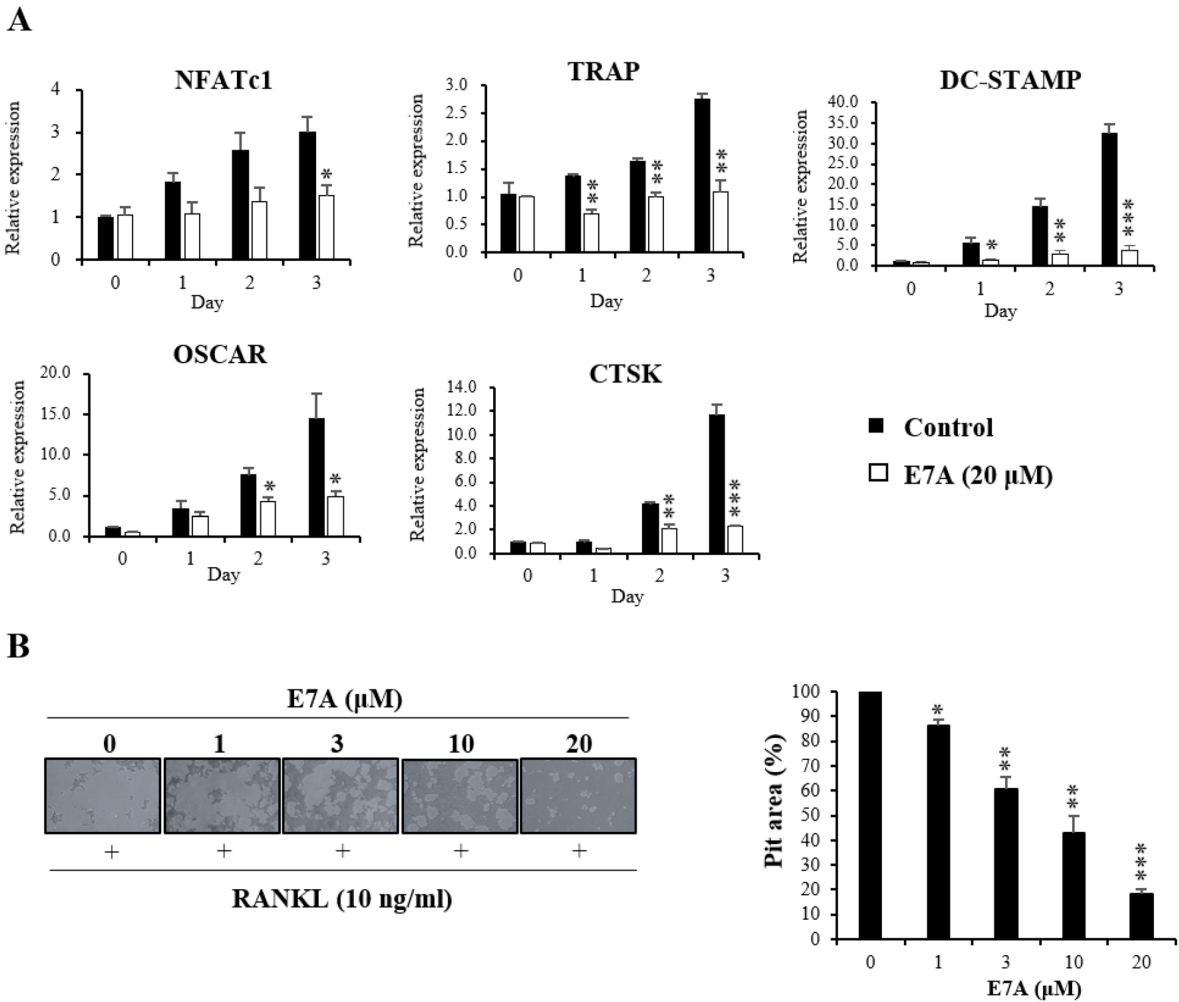
(**A**) Effect of E7A on RANKL-induced mRNA expression of osteoclast-specific genes. The mRNA expression levels of NFATc1, TRAP, DC-STAMP, OSCAR and cathepsin K (CTSK) were measured by real-time qPCR. GAPDH was used as an internal control. (**B**) Resorption pit assay for osteoclasts. BMCs (6 × 10^4^ cells/well) were cultured in a 24-well tissue culture plate in α-MEM containing 30 ng/mL M-CSF with or without 10 ng/mL RANKL in the absence or presence of E7A (1, 3, 10 or 20 μM) for 4 d. On day 4, after removing the medium, 100 µL of 10% bleach solution was added. The resorption region was observed with a light microscope (100 × magnification) and measured by Image J software. Data are mean ± SD (n = 3). *P < 0.05, **P < 0.01, ***P < 0.001 versus the 0 day by Student’s t-test. Scale bar, 500 μm.

Inhibition of gene expression involved in RANKL-induced osteoclast differentiation by E7A was analyzed using real-time qPCR. When RANKL-activated BMCs were treated with 20 µM E7A, the mRNA expression of NFATc1, TRAP, CTSK, OSCAR and DC-STAMP was significantly suppressed from day 1 and 3 (Fig. 3A). In addition to real-time qPCR of mRNA expression of various genes involved in osteoclast differentiation, we performed resorption pit assay to investigate the effect of E7A on the bone resorption function of mature osteoclasts. The extent of the pits decreased in an E7A concentration-dependent manner in RANKL-treated BMCs (Fig. 3B).

### Optimization of E7A-rich *Um*Hb extract through response surface methodology

Four independent variables (temperature, pH, time, and solvent ratio) were considered to determine the best conditions for obtaining the E7A-rich *Um*Hb extract. RSM was used to statistically analyze the interaction effects of multiple independent variables on the response^14^, and the second-order polynomial equation was utilized:

$$\begin{aligned} {\text{E7A }} = & { 8}.{64} + {1}.{\text{23X}}_{{1}} + {5}.{\text{86X}}_{{2}} - {4}.{\text{48X}}_{{3}} + 0.{\text{43X}}_{{4}} + {1}.{\text{78X}}_{{1}} {\text{X}}_{{2}} - 0.0{\text{58X}}_{{1}} {\text{X}}_{{3}} + 0.{\text{45X}}_{{1}} {\text{X}}_{{4}} - {7}.{\text{13X}}_{{2}} {\text{X}}_{{3}} \\ & - \;0.{4}0{\text{X}}_{{2}} {\text{X}}_{{4}} + 0.{\text{39X}}_{{3}} {\text{X}}_{{4}} - 0.{\text{71X}}_{{1}}^{{2}} + {1}.{\text{45X}}_{{2}}^{{2}} - {3}.{\text{85X}}_{{3}}^{{2}} - {1}.{\text{15X}}_{{4}}^{{2}} \\ \end{aligned}$$

Table 1 indicates the analysis of variance (ANOVA) of the RSM models for the response. The results showed that the quadratic model was significant (*P* < 0.05) with a non-significant lack of fit (*P* > 0.05).

**Table 1:** ANOVA for the quadratic models for the response.

Source	Sum of squares	Df	Mean square	F-value	*P*-value
Model	1025.30	14	73.24	5.73	0.0012
X_1_	18.28	1	18.28	1.43	0.2518
X_2_	412.46	1	412.46	32.25	< 0.0001
X_3_	241.29	1	241.29	18.86	0.0007
X_4_	2.27	1	2.27	0.18	0.6800
X_1_X_2_	12.62	1	12.62	0.99	0.3374
X_1_X_3_	0.013	1	0.013	0.00104	0.9747
X_1_X_4_	0.82	1	0.82	0.0639	0.8041
X_2_X_3_	203.59	1	203.59	15.92	0.0013
X_2_X_4_	0.65	1	0.65	0.0507	0.8250
X_3_X_4_	0.60	1	0.60	0.047	0.8313
X_1_^2^	3.23	1	3.23	0.25	0.6234
X_2_^2^	13.67	1	13.67	1.07	0.3188
X_3_^2^	96.33	1	96.33	7.53	0.0158
X_4_^2^	8.57	1	8.57	0.67	0.4269
Residual	179.08	14	12.79		
Lack of fit	160.72	10	16.07	3.50	0.1192
R^2^	0.85				
Adj R^2^	0.70				

**Figure 4:**
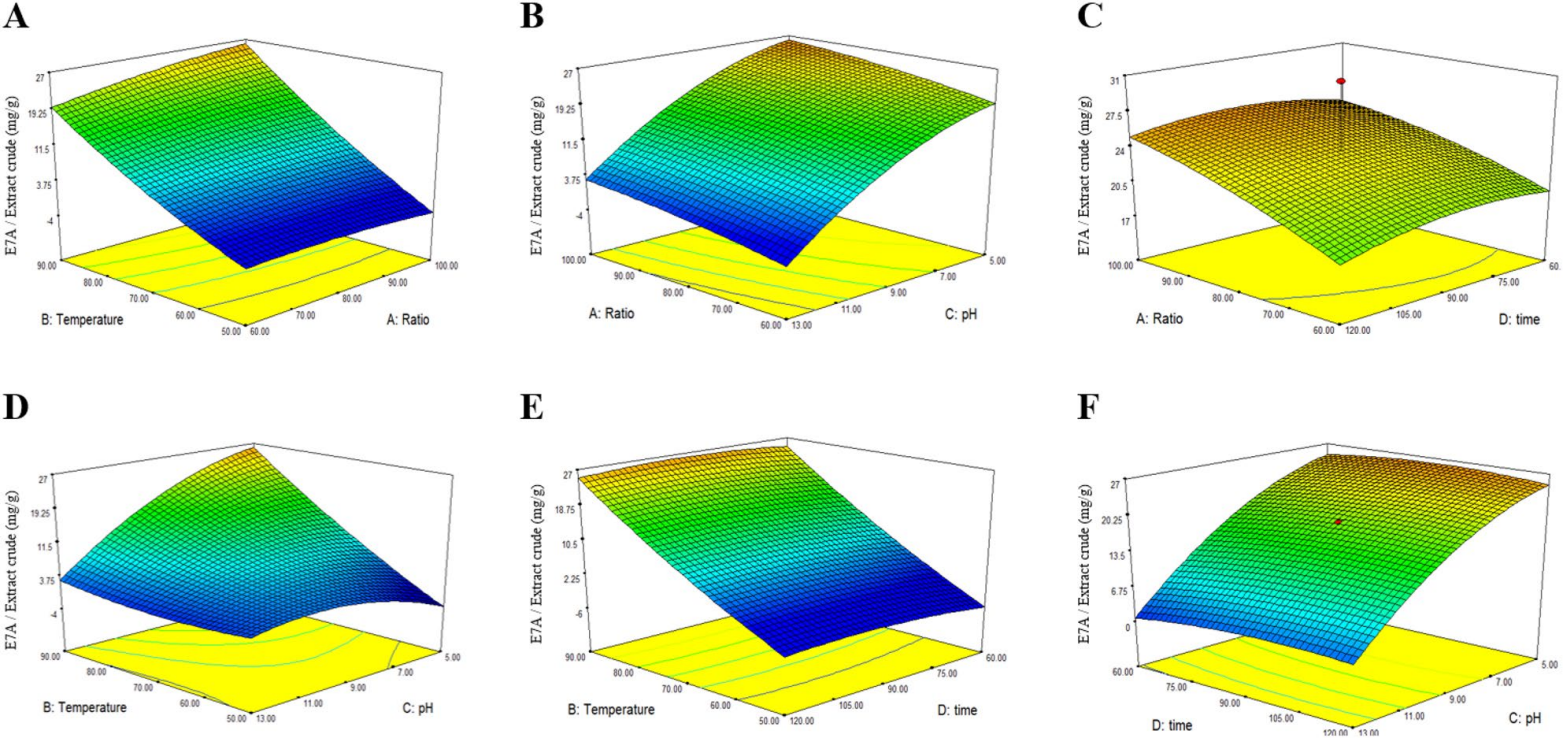
Three-dimensional surface plots showing the effects of solvent-to-solid ratio, temperature, pH, and extraction time on E7A content in *Um*Hb extracts. (**A**) Measured the amount of E7A with ratio and temperature as the parameters. (**B**) Ratio and pH, (**C**) ratio and time, (**D**) temperature and pH, (**E**) temperature and time, and (**F**) time and pH as independent variables.

The effects of the extraction parameters on the E7A content from *Um*Hb extracts are represented in Fig. 4 with a three-dimensional response surface diagram. The optimum extraction conditions to maximize the content of E7A in *Um*Hb extract were 100 mL/g, 90 °C, pH 5, and 97 min. The yield of E7A was predicted to be 26.05 mg/g extract with a desirability value of 0.856. The triplicate experiments verified the predicted condition with the value of 26.05 ± 0.96 mg E7A/g extract.

### Inhibition of RANKL-induced NFATc1 activation by the optimized *Um*Hb extract

**Figure 5:**
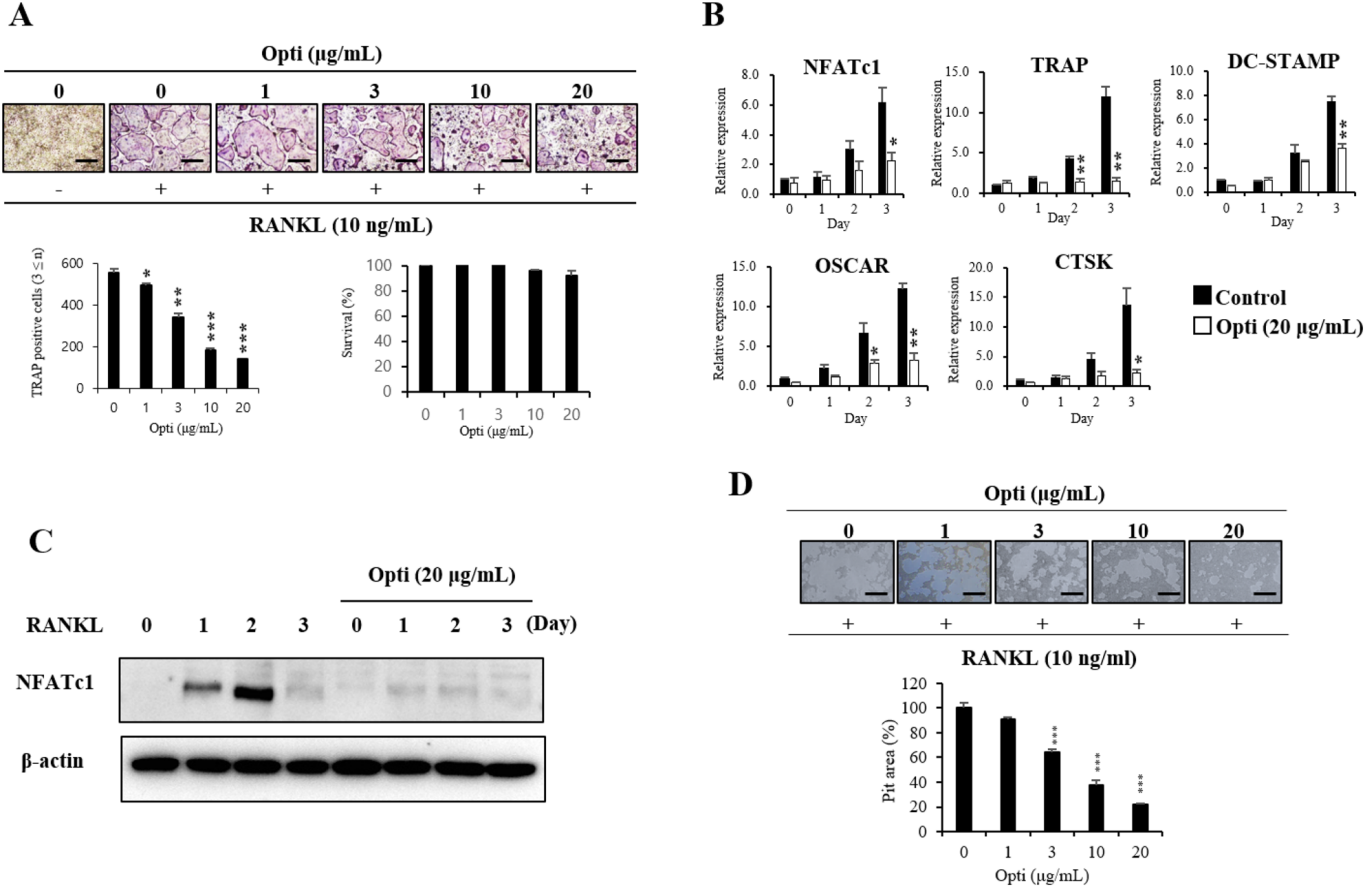
(**A**) RANKL-mediated osteoclastogenesis was suppressed by optimized *Um*Hb extracts, which had no cytotoxic effects. TRAP-positive MNCs (nuclear number > 3) were counted (n = 3). (**B**) Effects of the optimized *Um*Hb extract on the RANKL-induced mRNA expression of osteoclast-specific genes. (**C**) Effect of the optimized *Um*Hb extract on the protein expression of the osteoclast-specific transcription factor NFATc1. (**D**) Resorption pit assay for osteoclasts. BMCs were plated at tissue culture plate and cultured in α-MEM containing 30 ng/mL M-CSF with or without 10 ng/mL RANKL in the absence or presence of optimized *Um*Hb extracts (1, 3, 10, or 20 μg/mL) for 4 d. On day 4, after removing the medium, 100 µL of 10% bleach solution was added. The resorption region was observed with a light microscope (100 × magnification) and measured by Image J software. Data are mean ± SD (n = 3). *P < 0.05, **P < 0.01, ***P < 0.001 versus the 0 days by Student’s t-test. Scale bar, 500 μm.

To investigate the inhibitory effect of optimized *Um*Hb extract on RANKL-induced osteoclast differentiation, BMCs were treated with M-CSF and RANKL in the absence or presence of the extract. TRAP staining, real-time qPCR, western blotting, and pit assays were performed (Fig. 5). According to the TRAP staining results, osteoclast differentiation was inhibited in proportion to the concentration of the extract, and differentiation into TRAP+MNCs was significantly reduced by the extract treatment. The mRNA expression of genes related to osteoclast differentiation was also investigated. Treatment of BMCs with 20 µg/mL extract significantly reduced the mRNA expression of NFATc1, TRAP, DC-STAMP, CTSK, and OSCAR (Fig. 5B). In addition, the extract significantly reduced the protein expression of *NFATc1* (Fig. 5C), a master regulator of *osteoclast* differentiation^15^.

## Discussion

Previously, we published that an ethanol extract of *Um*Hb exhibits anti-osteoclastogenic activity^12^. As *Um*Hb has been traditionally consumed as tea and used as a herbal medicine for an extended period, we hypothesized that it would also be effective in hydrothermal extracts. We confirmed its effectiveness in inhibiting osteoclast differentiation according to the extraction solvent conditions. As a result, investigated the effect of inhibiting osteoclast differentiation was better in hydrothermal extracts, and we hypothesized that, unlike ethanol extracts, specific compounds would be present in hydrothermal extracts (Fig. 1A). By comparing the HPLC data of the three types of extracts, a peak (15.53 min) was found only in hydrothermal extracts, and it was expected that the substance present at this location would have the specific osteoclast differentiation inhibition effect in hydrothermal extracts (Fig. 1B).

We conducted a comparative experiment with E7A, a specific compound of *Um*Hb hydrothermal extracts and UA, the most effective compound in the ethanol extract, and catechin, a representative backbone structure of *U. macrocarpa* Hance bark flavonoid. As shown in the result data, E7A showed a superior performance compared to other compounds, representing to be a key substance in inhibiting osteoclast differentiation (Fig. 2A). Taken together, these results indicated that E7A effectively inhibited bone resorption function and the regulation of NFATc1 expression is an important mechanism for this action of E7A. In addition, catechin (a common backbone structure) was not effective in inhibiting osteoclast differentiation. But ulmoside A, a glycoside attached catechin have effects although less effective than E7A. It means glycoside residue is also expected to play a very important role in efficacy.

And comparing flavan-3-ol((−)-epigallocatechin, (−)-epigallocatechin gallate, and (−)-epicatechin gallate (EGC)), which is effective on osteoclast differentiation of green tea and E7A with structure–function relationship, 2nd and 3rd carbon at flavan-3-ol have a common point of (R, R) configuration. Among the flavonoids of green tea extracts, EGC was the most potent in the inhibition of osteoclast differentiation, and (−)-epigallocatechin and (−)-epigallocatechin gallate have a relatively small effect^16^. While the carbon bond is (R, S) configuration, it was confirmed that catechin was ineffective in this study. In addition, the past studies suggest that the efficacy of catechin-7-O-D-apiofuranoside consists of (R, S) configuration -only variants, less than E7A, which consists of (R, R) configuration structures^12^.

E7A is a structure containing epicatechin of (R, R) configuration flavan-3-ol structures, showing a similar IC50 of inhibitory effect to EGCG(6.6 mM), which has the best osteoclast differentiation inhibition effect of green tea, which is consistent with the above logic^17^. By listing the above results, it can be confirmed that the (R, R) configuration binding structure plays a key role in inhibiting osteoclast differentiation. It was therefore expected that the osteoclast differentiation inhibitory effect would be affected not only by the backbone structure of catechins but also by the location or type of additionally attached glycosides.

In the realm of natural product studies, it is often observed that species within the same Genus share similar biosynthetic mechanisms. With this in mind, we investigated whether *Um*Hb is the optimal choice for extracting E7A. We collected five types of the *Ulmus* genus, which are native to Korea, and analyzed their E7A content under the same extraction conditions. E7A was detected in small amounts in most trees, with the highest concentration found in *Ulmus macrocarpa* Hance. Based on these results, we tentatively concluded that *Um*Hb is the most suitable material for achieving high purity and efficient extraction of E7A. It is difficult to conclude the effect of inhibiting osteoclast differentiation of E7A only with TRAP assay, and this was proved through bioassay in various methods. NFATc1, TRAP, DC-STAMP, OSCAR, and CTSK were selected as the genes to be confirmed. Bone metabolism is achieved through a complementary balance between the osteoblasts that make the bone matrix and the osteoclasts that absorb bone^18^. Osteoclasts are generated by proliferation, differentiation, fusion, and activation, starting with hematopoietic stem cells^19,20^. The cytokines macrophage colony-stimulating factor (M-CSF) and receptor activators of nuclear factor-κB ligand (RANKL) released by osteoblasts play essential roles in the differentiation and generation of osteoclasts in hematopoietic stem cells^21^. In the presence of M-CSF, the interleukin-1 mediated autocrine mechanism allows osteoclasts to interact with bone matrix^22^, ultimately leading to the increased plasma calcium level. The released RANKL binds to its receptor RANK which is expressed on the surface of osteoclast precursor cells via M-CSF stimulation. When osteoclast precursor cells with their surface receptor (RANK) bind to RANKL, these cytokines activate important signals indicative of osteoclast activity by various adaptor proteins and kinases^23,24^.

In the early stages of RANK signaling, RANKL is recruited to the cytoplasmic tail of RANK through adaptor proteins such as TRAF6 (the major adaptor protein responsible for mediating signaling cascades that are activated by RANKL)^25^, which leads to the rapid activation of MAPKs, NF-κB, and activator protein-1 (AP-1)^26^. In the next step of the signaling process, cytoplasmic 1 (NFATc1), which is a nuclear factor of activated T cells, and a key osteoclastogenesis regulator, is amplified by the orchestrated signaling of activated AP-1 and costimulatory signal-mediated intracellular Ca^2+^ oscillation^27^. As a final step, the osteoclastogenic gene transcribed by NFATc1 serves as a mediator to regulate multinucleation and bone absorption in the nucleus^24^. In a previous study, we found that flavonoid glycosides in the *Um*Hb ethanol extract inhibited RANKL and M-CSF-induced osteoclast differentiation in BMCs through the expression of NFATc1^12^. NFATc1 is an important transcription factor and regulates the expression of numerous target genes for osteoclast differentiation including TRAP, Cathepsin K (CTSK), osteoclast-associated receptor (OSCAR), and dendritic cell-specific transmembrane protein (DC-STAMP)^15^. TRAP is a highly expressed protein in osteoclasts that can be used as an indicator of osteoclast formation^28^. CTSK, which is involved in bone resorption, is responsible for the degradation of type I collagen during osteoclast-mediated bone resorption^29^. DC-STAMP and OSCAR are involved in the multinucleation process by cell–cell fusion of mononuclear osteoclasts during osteoclast differentiation^30,31^.

In the PCR technique, the result that E7A inhibits osteoclast differentiation compared to the control was confirmed in NFATc1, TRAP, DC-STAMP, OSCAR, and CTSK. This result provided a clue that differentiation was suppressed not only in TRAP assay but also in genetic levels (Fig. 3A). Since these factors are also used as indicators to confirm osteoclast differentiation inhibitory ability in various in vivo experiments, the possibility to have positive results in animal experiments as well in in vitro can be expected. The osteoclasts destroy bone tissue, forming a three-dimensional hole in the bone surface, called the “resorption pit” in vitro*.* Studies on osteoporosis have investigated the degree of absorption using the size and depth of the resorption pit^32^. In addition, it was confirmed that E7A has a concentration-dependent effect of inhibiting osteoclast differentiation even in pit assay. In the differentiation of osteoblasts, the activity was slightly increased when BMP-2 (100 ng/mL) was treated with E7A (Supplementary Fig. S10). As a result, E7A inhibits differentiation in osteoclasts independently of the osteoblast mechanism.

To obtain the most efficient E7A-rich extract, pH, temperature, ratio, and time was arrayed as independent variables. Among the variables, the effects of temperature and pH, as well as the interaction between them, were found to be significant, with a coefficient of determination (R^2^) of 0.8513. An increase in temperature leads to an increase in the diffusion coefficient and solubility of flavonoids, as well as a significant increase in solvation and mass transfer^33^. Moreover, the elevated temperature can trigger the release of flavonoids from plants through the breakdown of cell structures^34^. Acidic conditions have been reported to affect the extraction efficiency of polyphenols such as anthocyanidin 3-glucosides^35,36^. We verified that the optimized E7A-rich extract of *Um*Hb showed the inhibition ability to osteoclast differentiation. The extracts had no cytotoxic effect on BMCs at the concentrations used in this experiment (1–20 µg/mL), indicating that the inhibition of osteoclast differentiation was not due to the cytotoxicity of the extract (Fig. 5A). Optimized extracts showed a significant osteoclast differentiation inhibition effect at both protein and mRNA level (Fig. 5B, C). The resorptive pit assay revealed that the extract reduced the extent of the pit in a dose-dependent manner (Fig. 5D). Taken together, these results suggest that the optimized *Um*Hb extract reduces osteoclast differentiation and the bone resorption function of mature osteoclasts through the downregulation of NFATc1 expression. *Um*Hb promotes osteoblast differentiation by BMP-2. It can be seen that the activity increases at a concentration of 20 µg/mL (Supplementary Fig. S10). Comparing the IC_50_ values of the *Um*Hb hydrothermal extract (calculated from data in Fig. 1A) and the optimized hydrothermal extract (Fig. 5), the IC_50_ was decreased from 3.57 to 3.17 µg/mL. These results indicate that the osteoclast differentiation inhibitory effect was enhanced by increasing the content of the active compound E7A through our optimization process (Supplementary Fig. S8). These E7A-rich extracts show potential as supplement improvement/treatment in bone-related diseases.

## Conclusion

**Figure 6:**
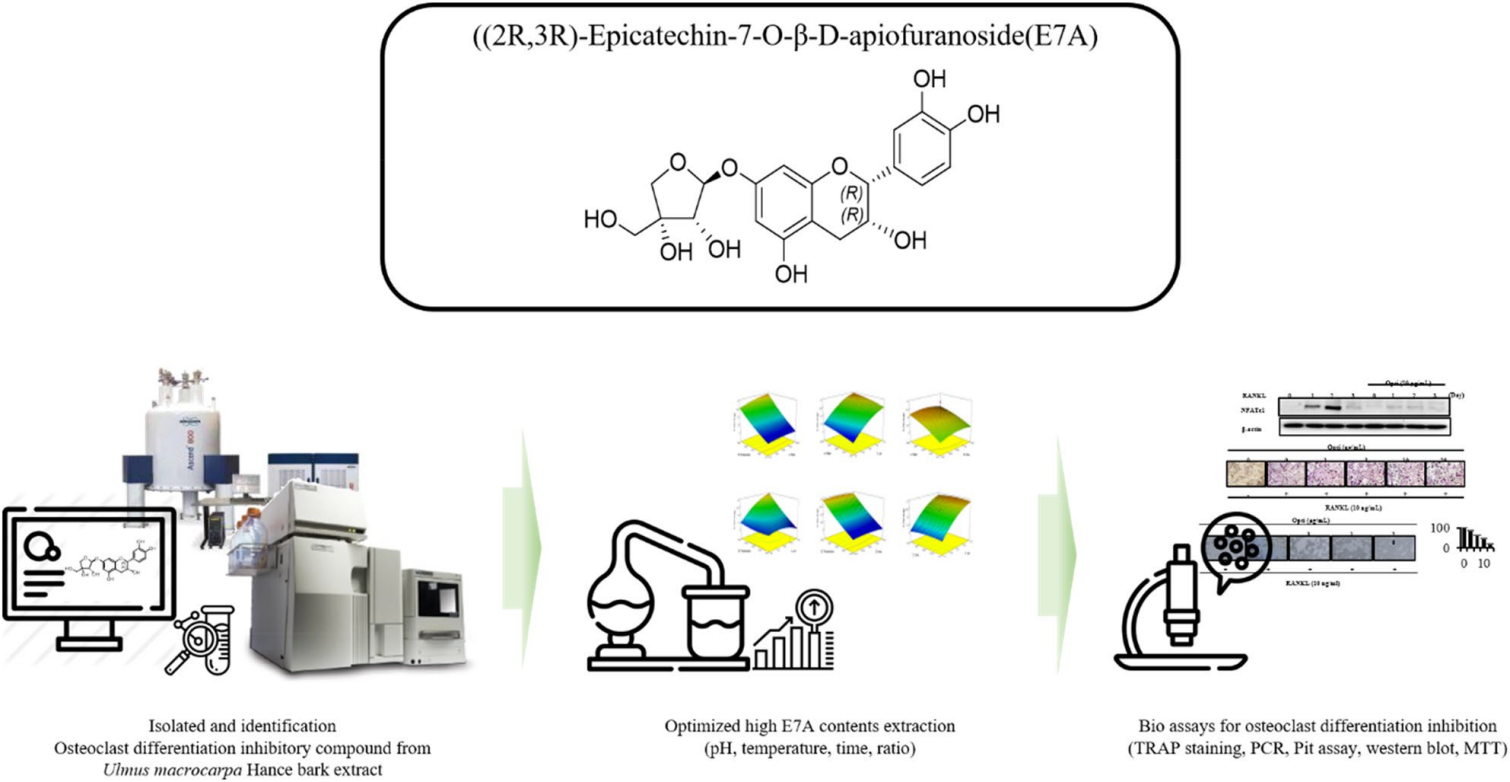
Summarize the whole study.

This study investigated the efficacy of water extracts as humans have traditionally drunk *Um*Hb tea as herbal medicine for many years. *Um*Hb hot water extract was more effective in inhibiting osteoclast differentiation than ethanol extract. (2R,3R)-epicatechin-7-O-β-D-apiofuranoside (E7A) was identified and purified as a substance sufficiently present in the water extract and was rarely seen in 70% and 100% ethanol extracts. The glycoside flavonoid E7A significantly inhibited RANKL-induced osteoclast differentiation, and the inhibitory effect was greater than that of ulmoside A, the most effective compound in the ethanol extract of *Um*Hb. To improve the extraction of E7A, we established optimal extraction conditions for *Um*Hb using RSM. Our results indicate that E7A and its optimal extract abrogate osteoclast differentiation through the inhibiting the expression of NFATc1 as well as other target genes related to osteoclast differentiation. The findings of our research have been encapsulated in a flow chart, presented as Fig. 6. These results indicate that E7A and its optimal extract have the potential to be developed as substances that can improve/prevent osteopenia disease. Future in vivo preclinical and clinical studies are needed to investigate whether and the mechanism by which E7A and optimal *Um*Hb extract inhibit osteopenia disease.

## Materials and methods

### General experimental procedures

The ultraviolet (UV) spectrum was obtained by using a Hitachi JP/U-3010 UV spectrophotometer (Tokyo, Japan). All NMR spectra were obtained with a Bruker Ascend 700 spectrometer (Billerica Middlesex County, MA, USA) using MeOH-d4 (Gif-sur-Yvette, France) as the solvent. NMR solvents were purchased from the Cambridge Isotope Laboratory (Andover, MA, USA).

Chemical shifts were obtained by referring to each solvent peak (HH 3.31 and CC 49.0 for methanol-d4). Low-resolution LC–MS data were acquired with an Agilent Technologies 1200 series HPLC (Agilent Technologies, Santa Clara, CA, USA) connected to an Agilent Technologies 6130 quadrupole mass spectrometer. High-resolution MS data were recorded with a Triple TOF 5600 High-resolution mass spectrometer (AB Sciex, Foster City, CA, USA) with a quadrupole/TOF mass spectrometer. All mass data were obtained with the electron spray ionization method (ESI).

### Plant materials, extraction and isolation of compounds, and analytic assays

#### Plant materials

Dried *Um*Hb was purchased from a local company (Jinsung F.M.). *U. pumila* L, *U. parvifolia Jacq*, *U. davidiana Planchon,* and *U. davidiana var. japonica* (Rehder) *Nakai* were provided by Seoul National University Forests (SNUF), and their identities were confirmed by one of the coauthors of this paper, Ki Won Lee^12^. This study complies with relevant legislation and international, national, and institutional guidelines. The plant material was dehydrated in a drying chamber under a controlled temperature at 50 °C, relative humidity, and air velocity to ensure the quality of the final product. The dried bark was trimmed into small pieces and ground into a fine powder using a blender. It was then handpicked and reduced to a fine powder (~ 20 mesh). The powdered samples were then transferred to an appropriately labeled polypropylene zipper bag and kept at − 80 °C for a maximum of 30 weeks.

#### Extraction of the bark of the genus *Ulmus*

Three hundred milligrams of powdered samples were added to 30 mL of distilled water at 70 °C and extracted for 120 min after being suspended. The extracted solution was centrifuged at 3000 × g for 1000 s at room temperature and the supernatant was filtered through Whatman filter paper No. 4.

#### Compound isolation

Dried *Um*Hb powder (50 g) was extracted with deionized water at 70 °C for 3 h. The dried water extract (312.5 mg) was resuspended in distilled water (250 mL), and the solution was sequentially fractionated with dichloromethane (250 mL), ethyl acetate (250 mL), and n-butanol (250 mL). The n-butanol layer was subjected to Sephadex LH-20 size exclusion chromatography and eluted with methanol to obtain 46 fractions (UMB1-UMB46). Fractions UMB13-46 contained similar chemical components. Thus, fraction UMB13-46 were combined and purified by RP-HPLC (Phenomenex Luna C18, 5 μm, 100 Å, 250 × 100 mm), eluting with 35% MeOHand only one compound was isolated (RT: 15.53 min; 13.5 mg) in the water extract.

*(2R,3R)-epicatechin-7-O-β-D-apiofuranoside (E7A)* white and brown amorphous powder; [α]20D -19.6 (c 0.52, MeOH); UV (MeOH) λ max (log ε) 203, 278; 1H NMR (methanol-d4, 700 MHz) δ 6.98 (d,J = 1.7 Hz, H-2′), 6.80 (dd, J = 8.1, 1.7 Hz, H-6′), 6.76 (d,J = 8.1 Hz, H-5′), 6.14 (d, J = 3.8 Hz, 1H, H6), 6.14 (d, J = 3.8 Hz, 1H, H8),4.82 (br s, H-2), 4.19 (m, H-3), 2.88 (dd, J = 16.8, 4.4 Hz, H-4a), 2.74 (dd, J = 16.8,2.6 Hz, H-4b), 2.88 (H-4a), 2.74 (H-4b), 2.86 (H-4a), 2.54 (H-4b), 5.49 (d, J = 2.9 Hz, H-1″), 4.14 (d, J = 2.9 Hz, H-2″), 4.09 (m, H-4″a), 3.85 (d, J = 9.7 Hz, H4″b), 3.62 (m, H-5″), 5.49 (d, J = 2.9 Hz, H-1″); 13C NMR (methanol-d4, 175 MHz) δ 108.72 (C-1″),79.94 (C-3″), 78.27 (C-2″), 75.40 (C-4″), 64.96 (C-5″), 158.01 (C-7), (C-C1′), 119.36 (C-6′), 115.90 (C-5′), 115.28 (C-2′), 102.51 (C-10), 97.40 (C-6), 97.17 (C-8), 80.29 (C-2), 67.29 (C-3), 29.27 (C4); ESI MS m/z 423 [M + H] + , 421 [M − H]−.

#### HPLC analysis

The extract and compounds were analyzed using a Waters HPLC system (Milford Worces County, MA, USA) equipped with a Waters 2998 photodiode array detector on an HPLC Phenomenex Luna C18 column (5 μm, 100 Å, 250 × 100 mm^2^). The temperature of the column was maintained at 24 °C. The chromatograms were processed at 210 nm, and the spectra were obtained between 210 and 400 nm. A gradient solvent system consisting of solvent A (0.1% (v/v) aqueous formic acid) and solvent B (0.1% (v/v) formic acid in acetonitrile) was used at 1 mL/min as following procedure: 0–2 min, 2 to 25% B; 2–20 min, 50 to 95% B. HPLC-grade solvents (Daejeon, Korea) were used for HPLC analysis.

### Biological analysis of isolated compounds

#### Osteoclast differentiation and tartrate-resistant acid phosphatase (TRAP) staining

Osteoclast differentiation and TRAP staining were performed as previously reported^12,13^. We isolated bone marrow cells (BMCs) according to Suncheon University's Animal Research Standard Protocol (SCNU). The Animal Use Protocol was approved by the Agency Animal Care and Use Committee of SCNU (Permit No. SCNU IACUC 2021-05). Briefly, BMCs were isolated by washing the femurs and tibias of a 5-week-old ICR mouse with PBS^20^. The isolated BMCs were cultured in α-MEM supplemented with 10% fetal bovine serum (FBS) (Invitrogen Life Technologies, Grand Island, NY, USA) with 10 ng/mL M-CSF (R&D Systems, Minneapolis, MN, USA) for 1 d. BMCs obtained by removal of the suspended cell debris in α-MEM were used for osteoclast differentiation. BMCs (1 × 10^4^ cells/well) were cultured with M-CSF (30 ng/mL) and RANKL (10 ng/mL, R&D Systems, Minneapolis, MN, USA) for 3 d to generate preosteoclasts. The preosteoclasts were treated with either vehicle or the indicated compounds in the presence of M-CSF (30 ng/mL) for 30 min and then cultured with RANKL (10 ng/mL) for differentiation into mature TRAP-positive multinucleated cells (TRAP+-MNCs). After one day, the cells were fixed with 3.7% formaldehyde for 5 min, permeabilized with 0.1% Triton X-100 for 5 min, and stained using the leukocyte acid phosphatase kit 387-A (Sigma Aldrich, St. Louis, MO, USA). It was confirmed that all the extract samples used in the experiment were fully dissolved to the maximum at a concentration of 10 mg/ml (Supplementary Fig. S9).

#### Cell viability assay

Cell viability was assessed by monitoring the conversion of 2-(2-methoxy-4-nitrophenyl)-3-(4-nitrophenyl)-5-(2,4-disulfophenyl)-2H-tetrazolium, monosodium salt (WST) to formazan according to the manufacturer's instructions (Dojindo, Kumamoto, Japan). Briefly, separated BMCs (1 × 10^4^ cells/well) were incubated in 96-well plates for 12 h in the presence of M-CSF (30 ng/mL) and then treated with the indicated concentrations of dimethyl sulfoxide (DMSO), extracts or flavonoid compounds. After 3 d, 10 μL of WST-8 solution was added to each well. The plate was incubated at 37 °C for 4 h, and then the absorbance was measured at 450 nm using a microplate reader (Thermo, Varioskan Flash, UK).

#### Real-time polymerase chain reaction (RT‒PCR)

Real-time PCR was performed as previously described^37^. BMCs were incubated with M-CSF (30 ng/mL) in 10% FBS α-MEM and activated with RANKL (10 ng/mL) for 0, 1, 2, or 3 d in the presence of E7A or the optimized *Um*Hb extract. PCR primer sets (Table 2) were designed using the online Primer3 program^38^. Total RNA was isolated using TRIzol reagent (Thermo Fisher Scientific Inc., Waltham, MA), and cDNA was synthesized using the M-MLV cDNA Synthesis Kit (Enzynomics, Daejeon, Korea). PCR was performed using a TOPreal qPCR 2 × PreMIX (Bio-Rad, Hercules, CA) in a real-time PCR detection system (Bio-Rad). The mRNA levels of the genes were determined using the $2^{{{-}\Delta \Delta C_{T} }}$ method. Glyceraldehyde-3-phosphate dehydrogenase (GAPDH) was used as an internal standard.

**Table 2:** Primer sequences were used in this study.

Primer sequence (5′ → 3′)		
Gene of interest	Sense	Antisense
NFATc1	GGGTCAGTGTGACCGAAGAT	GGAAGTCAGAAGTGGGTGGA
CTSK	GGCCAACTCAAGAAGAAAAC	GTGCTTGCTTCCCTTCTGG
OSCAR	CTGCTGGTAACGGATCAGCTC	CCAAGGAGCCAGAACCTT
DC-STAMP	CCAAGGAGTCGTCCATGATT	GGCTGCTTTGATCGTTTCTC
TRAP	GATGACTTTGCCAGTCAGCA	ACATAGCCCACACCGTTCTC
GAPDH	AACTTTGGCATTGTGGAAGG	ACACATTGGGGGTAGGAACA

#### Western blotting

Western blotting analysis was performed as previously described^37^. Briefly, BMCs were incubated with RANKL (10 ng/mL) and M-CSF (30 ng/mL) in 10% FBS α-MEM for 0, 1, 2, or 3 d in the presence of the optimized *Um*Hb extract. Harvested cells were lysed with a lysis buffer containing protease inhibitors and quantified using the Bradford assay. Isolated proteins were separated using 10% sodium dodecyl sulfate–polyacrylamide gel electrophoresis (SDS‒PAGE) and transferred to a polyvinylidene difluoride (PVDF) membrane (Millipore, Bedford, MA, USA). The membrane was incubated with a primary antibody (NFATc1) at 4 °C overnight. β-Actin was used as the loading control.

#### Resorptive pit assay

To analyze the inhibitory activities of E7A and the optimized *Um*Hb extract on the bone resorption function of mature osteoclasts, the surface for pit formation was analyzed. BMCs were plated at 6 × 10^6^ cells/well in 24-well tissue culture plates and cultured with 30 ng/mL M-CSF and 10 ng/mL RANKL in the presence or absence of E7A or the optimized *Um*Hb extract for 4 d. On day 4, after removing the medium, 100 µL of 10% bleach solution was added. After incubation for 5 min at room temperature, the plate was washed twice with distilled water and dried at RT for about 4 h. Individual pits or clusters of multiple pits were observed using a microscope at 100 × magnification.

#### Osteoblast assay

Mouse myoblast cell line, C2C12 was obtained from the American Type Culture Collection (Manassas, VA, USA). C2C12 cells were maintained in 10% FBS DMEM, 100 units/mL penicillin, and 100 µg/mL streptomycin. Cells were seeded in 96-well plates at 2.5 × 10^3^ cells/well. After 4 d, cells were differentiated by replacing the medium with 10% FBS DMEM and BMP-2 (100 ng/mL) with E7A, the optimized *Um*Hb extract at the indicated dose for 4 days. Osteoblastic bone formation was observed by ALP staining.

### Optimization of E7A-rich *Um*Hb extract

#### Experimental design and quantitation of the compound as a response

Heat-assisted extraction (HAE) optimization was performed on a thermostatic heater using a sealed vessel to prevent solvent evaporation. The independent variables employed to maximize the E7A concentration of the *Um*Hb extract were extraction time (t, 60–120 min), temperature (T, 50–90 °C), solvent pH (pH, pH 5–13), and solid/liquid ratio (S/L, 60–100%). The dry powder samples were mixed with 30 mL of solvent (distilled water) and processed under continuous electromagnetic stirring according to the experimental design presented in Table 1. After extraction, the mixture was centrifuged (3000 × g for 1000 s at room temperature) and the supernatant was filtered through Whatman filter paper No. 4. The concentration of E7A was calculated using purified E7A (purity 95.47%, Supplementary Fig. S10) in our laboratory as a standard, which is not commercially available in purified form.

#### Response surface methodology

Box‒Behnken design (BBD) with independent variables [X_1_ (t, min), X_2_ (T, °C), X_3_ (pH), and X_4_ (S/L)] was applied to optimize the extraction of E7A from *Um*Hb by HAE. The BBD, including 29 independent combinations with 5 replicates at the center of the experimental design, was chosen to maximize the predictive capacity of the model (Table 1). The predicted response (Y) was expressed as a second-order polynomial model with four independent variables using the following equation:

$$Y = \beta_{0} + \mathop \sum \limits_{i = 1}^{4} \beta_{i} X_{i} + \sum \mathop \sum \limits_{i < j = 1}^{4} \beta_{ij} X_{i} X_{j} + \mathop \sum \limits_{i = j}^{4} \beta_{ii} X_{i}^{2} ,$$

Where X_i_ and X_j_ represent the independent variables, and β_0_, β_i_, β_ij_ and β_ii_ represent the regression coefficients for the intercept, and linear, quadratic and interaction terms, respectively.

### Statistical analysis

The results are represented as the mean ± SD of three replicates. Statistical differences were analyzed using Student’s t-test. Probability values lower than 0.05 were considered to be significant (*P* values * < 0.05, ** < 0.01, *** < 0.001).

## Supplementary Information

Supplementary Information.

## Data Availability

All data generated or analyzed during this study are included in this published article and its supplementary information file.
