# Supplementary material for: Discovery and optimized extraction of the anti-osteoclastic agent epicatechin-7-O-β-D-apiofuranoside from Ulmus macrocarpa Hance bark
Source: Sci Rep. 2023 Jul 9;13:11102. doi: 10.1038/s41598-023-38208-4 (PMC10330169; doi:10.1038/s41598-023-38208-4)
Supplement: Supplementary file 1 — Supplementary Information. [file 41598_2023_38208_MOESM1_ESM.pdf]

**SUPPORTING INFORMATION**

Figure S1. Cytotoxicity data of *Um*Hb extracts (MTT).

Figures S2. 1H and 13C nuclear magnetic resonance (NMR) spectra based E7A from *Um*Hb water extracts.

Figure S3. Correlated spectroscopy (COSY) NMR spectrum of based E7A from *Um*Hb water extracts.

Figure S4. Heteronuclear single quantum coherence (HSQC) NMR spectrum of peak based E7A from *Um*Hb water extracts.

Figure S5. Heteronuclear multiple bond correlation (HMBC) NMR spectrum of peak based E7A from *Um*Hb water extracts.

Figure S6. Cytotoxicity data of ulmoside A, catechin and E7A from *Um*Hb extracts (MTT).

Figure S7. 100~600 M/Z(Da) parts of Genus *Ulmus* extracts Mass data at 15.53min. E7A are m/z 423 $[M + H]^+$, 421 $[M – H]^-$.

Figure S8. Comparison of the efficacy of *Um*Hb hydrothermal extracts in Figure 1A and optimized UmHb hydrothermal extracts in inhibiting osteoclast differentiation through TRAP staining.

Figure S9. *Um*Hb extracts dissolved maximum at a concentration of 10 mg/ml.

Figures S10. Osteoblast differentiation data of *Um*Hb, E7A by ALP staining

Figures S11.Purity data of laboratory purified E7A at 220nm (HPLC).

Figures S12. Optimization of the method in flow chart in this study

Figures S13. Uncropped western blotting images (Figure 5)

Table S1. Box-Behnken experimental design and response parameters for obtaining E7A-containing extracts from UmHb.

Figure S1. Cytotoxicity data of *Um*Hb extracts (MTT). (A) Cytotoxicity of *Um*Hb water extracts (B) Cytotoxicity of *Um*Hb 70% ethanol extracts (C) Cytotoxicity of *Um*Hb 100% ethanol extracts.

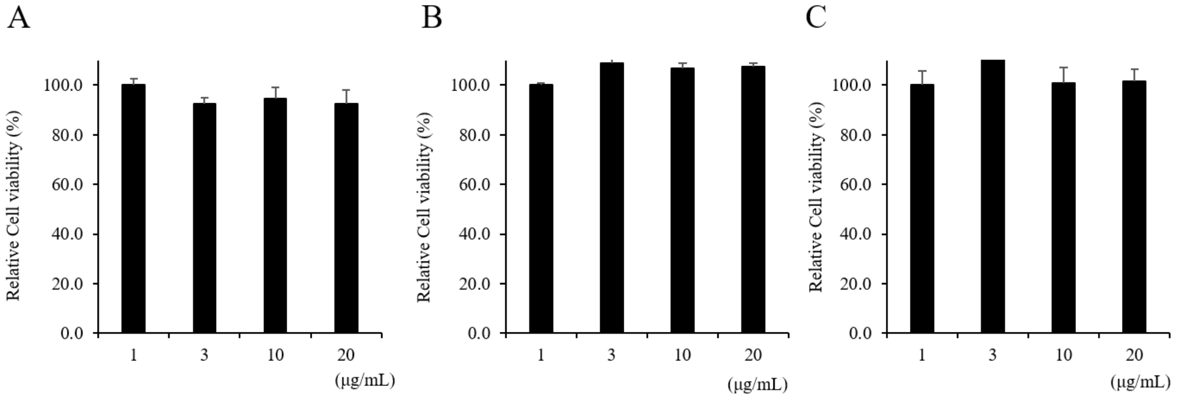

Figures S2. 1H and 13C nuclear magnetic resonance (NMR) spectra based E7A from *Um*Hb water extracts.

(A) 1H nuclear magnetic resonance (NMR) spectra based E7A (B) 13C nuclear magnetic resonance (NMR) spectra

Figure S1-A

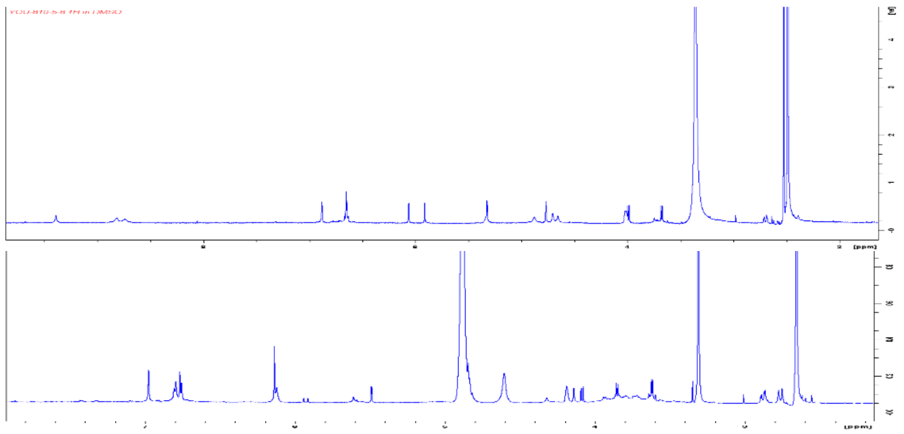

Figure S1-B

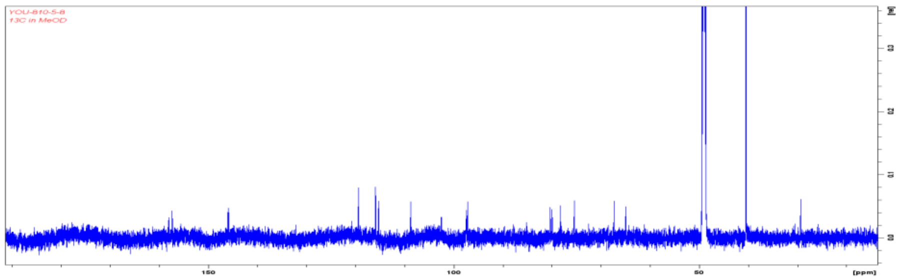

Figures S3. Correlated spectroscopy (COSY) NMR spectrum of based E7A from *Um*Hb water extracts.

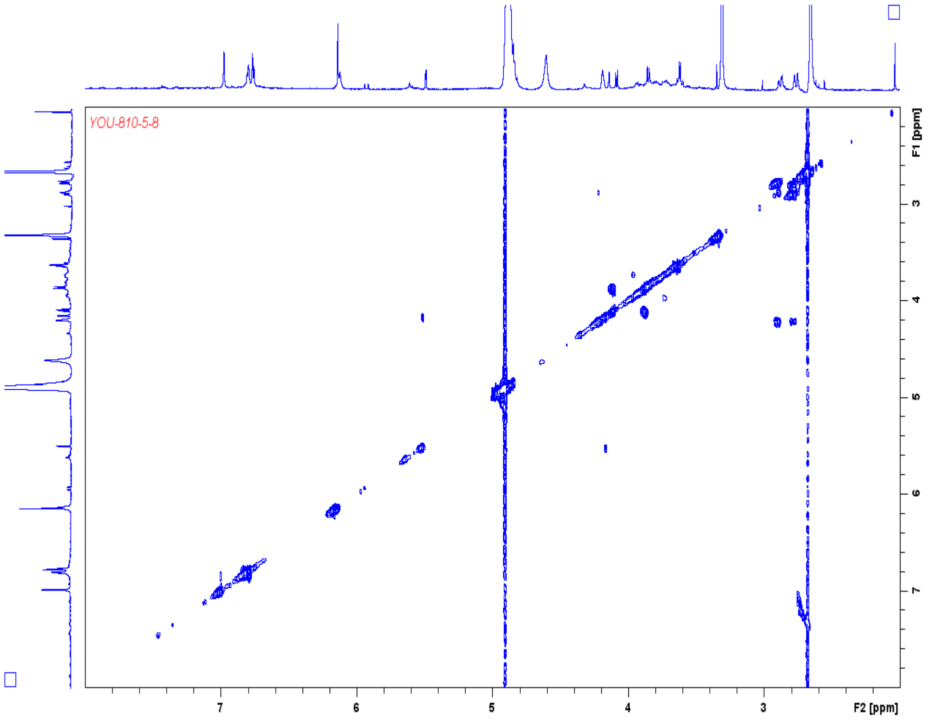

Figure S4. Heteronuclear single quantum coherence (HSQC) NMR spectrum of peak based E7A from *Um*Hb water extracts.

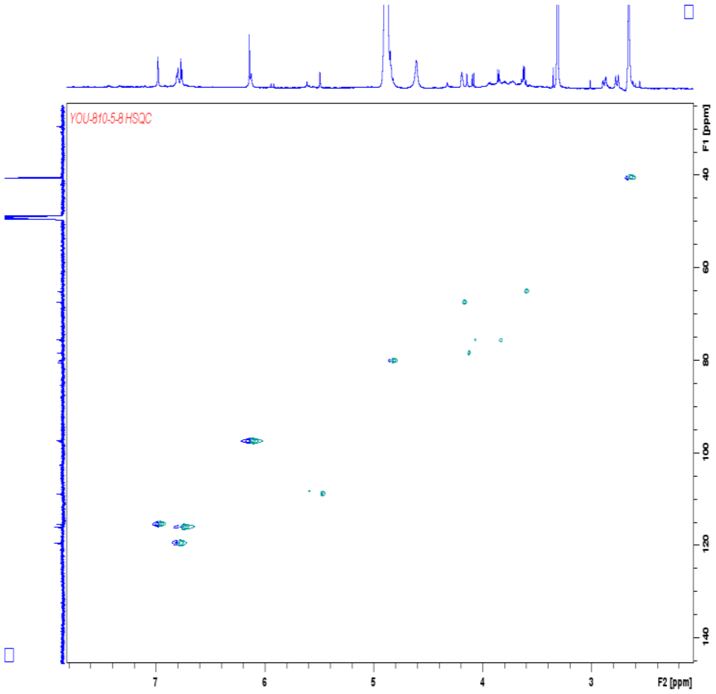


-

Figure S5. Heteronuclear multiple bond correlation (HMBC) NMR spectrum of peak based E7A from *Um*Hb water extracts.

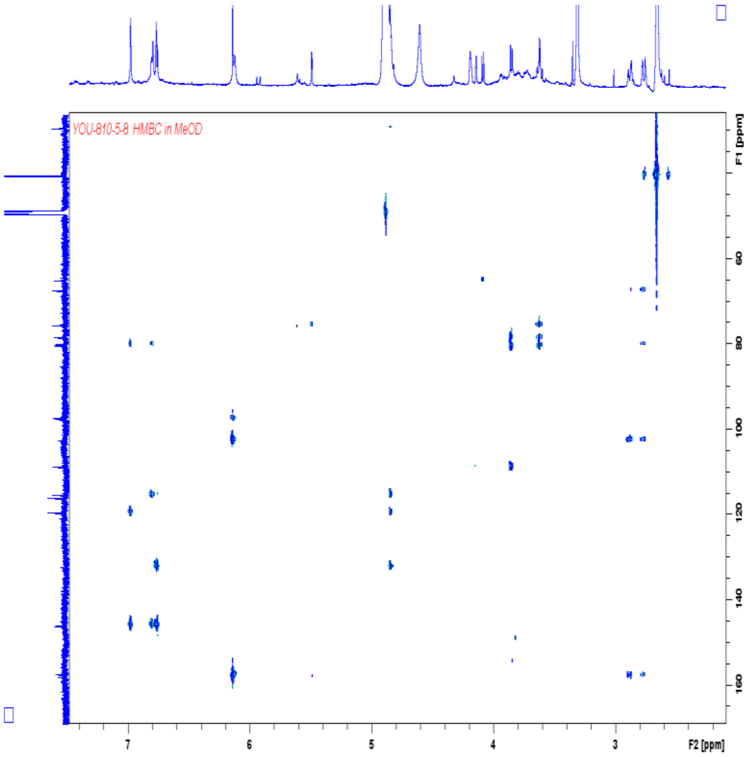

Figure S6. Cytotoxicity data of catechin, ulmoside A and E7A from *Um*Hb extracts (MTT).

(A) Cytotoxicity data of catechin (B) Cytotoxicity data of ulmoside A (C) Cytotoxicity data of E7A

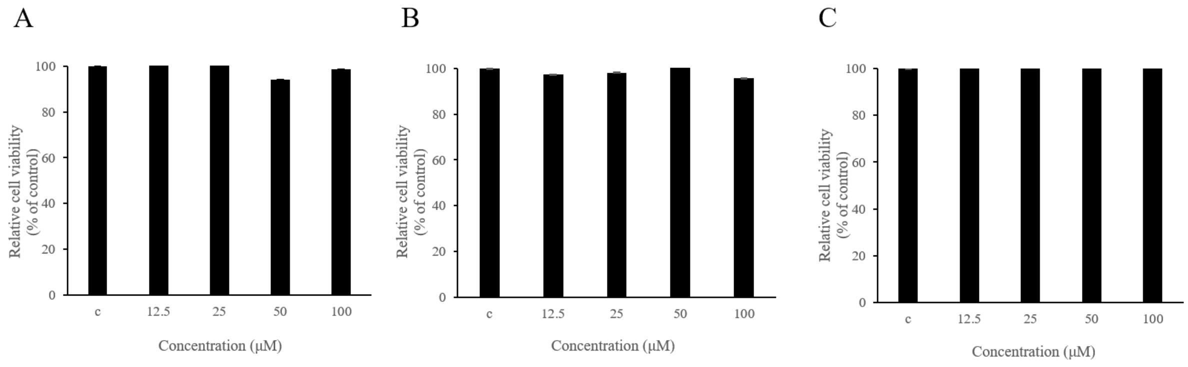

Figure S7. 100~600 M/Z(Da) parts of Genus *Ulmus* extracts Mass data at 15.53min. E7A are m/z 423 [M + H]+, 421 [M – H]-.

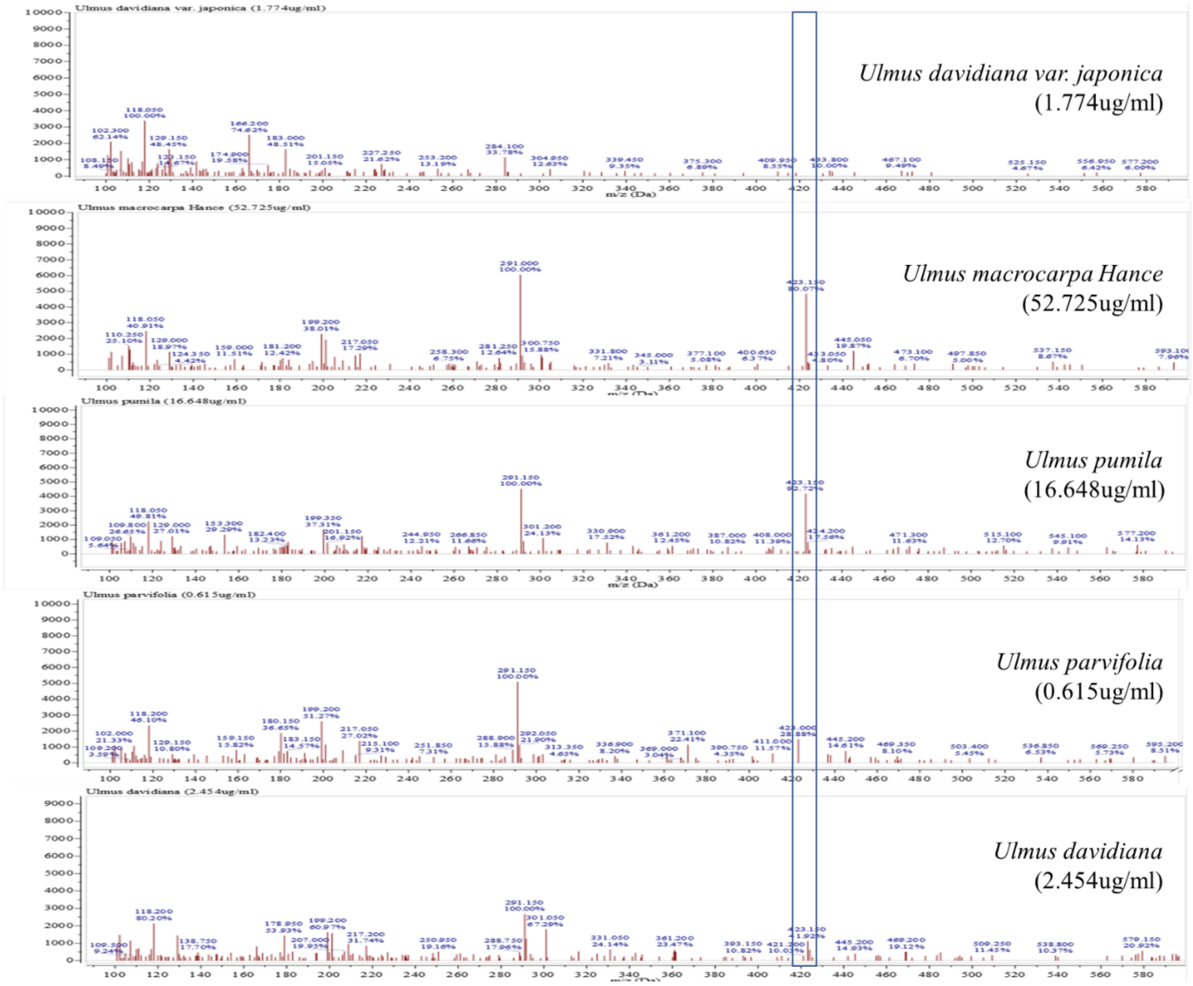

Figure S8. Comparison of the efficacy of *Um*Hb hydrothermal extracts in Figure 1 and optimized *Um*Hb hydrothermal extracts in inhibiting osteoclast differentiation through TRAP staining.

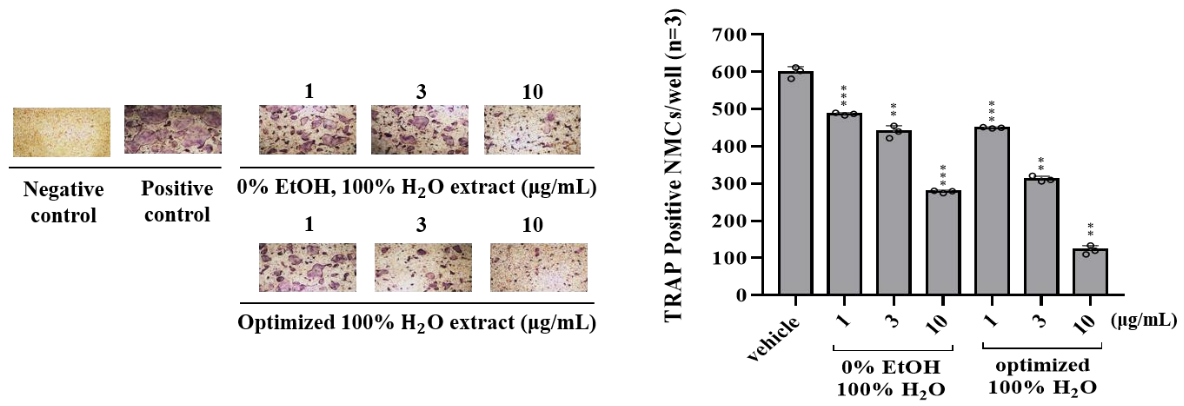

Figure S9. UmHb dissolved maximum at a concentration of 10 mg/ml

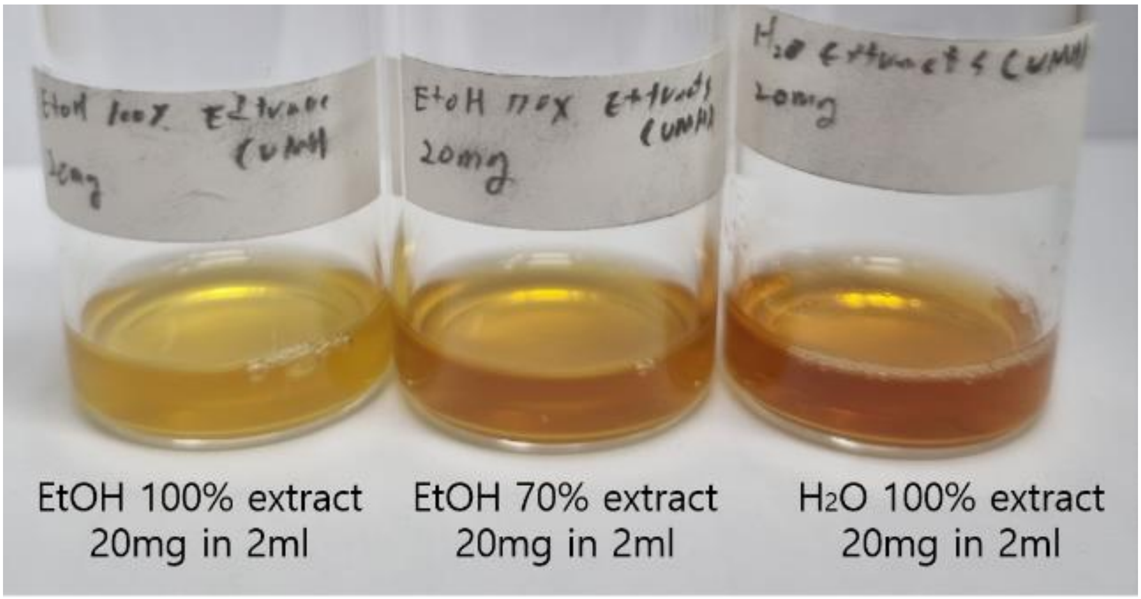


As specified in the paper, 30 ml of solvent(70% EtOH, 100% EtOH) was added to 300 mg of raw material and heated at 70°C for 120 minutes. The extracted samples were dissolved in sterile distilled water at a concentration of 10 mg/ml, and complete dissolution was observed.

Figures S10. Osteoblast differentiation data of UmHb , E7A by ALP staining

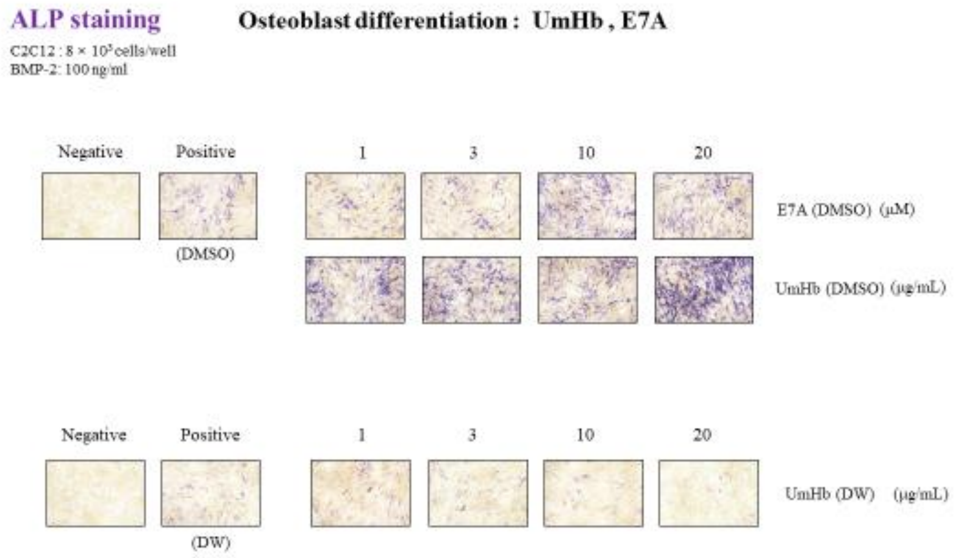


UmHb promotes osteoblast differentiation by BMP-2. C2C12 cells cultured in the presence of BMP-2 (100 ng/mL) at the indicated UmHb concentration for 4 days. Confirmation of osteoblast differentiation is measured by the intensity of alkaline phosphatase (ALP) staining.

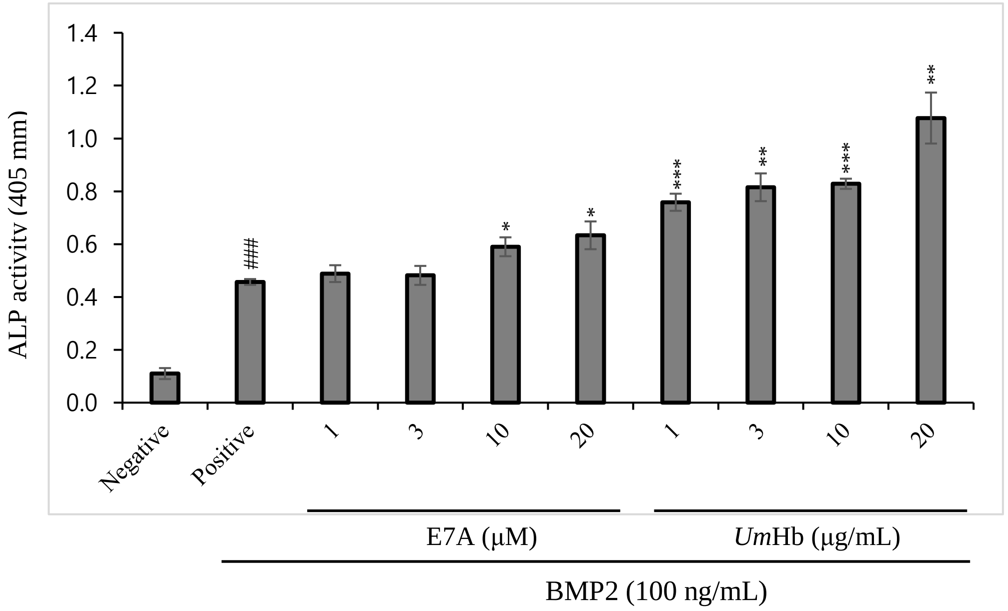


(#: versus negative group, *: versus positive group)

Figure S11. Purity data of laboratory purified E7A at 220nm (HPLC).

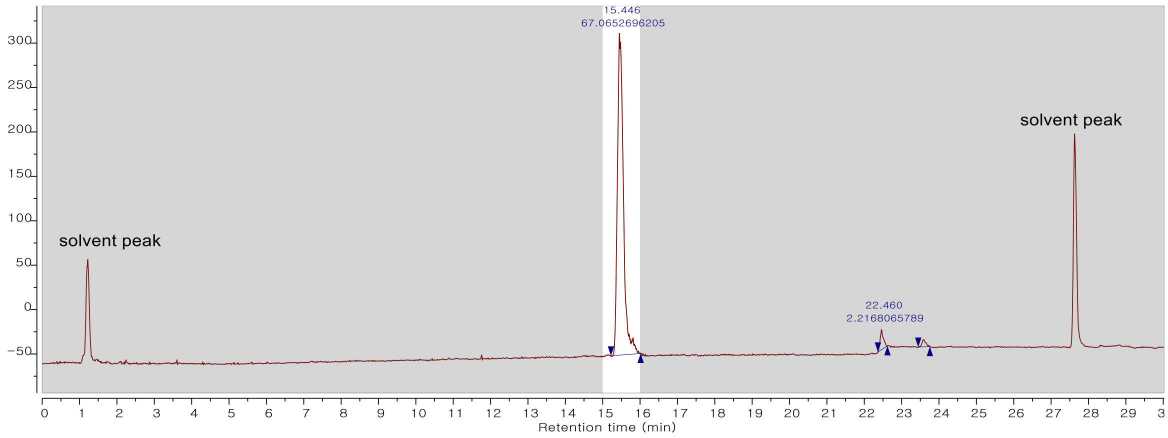


| | RT | Height | Area | Total Height % | Total Area % | Start time | End time |
|---|---|---|---|---|---|---|---|
| Mass Peaks | | | | | | | |
| 1 | 23.575 | 8.307 | 0.966 | 2.11 | 1.38 | 23.444 | 23.755 |
| 2 | 22.460 | 22.840 | 2.217 | 5.80 | 3.16 | 22.365 | 22.620 |
| 3 | 15.446 | 362.585 | 67.065 | 92.09 | 95.47 | 15.221 | 16.016 |

Figures S12. Optimization of the method in flow chart in this study

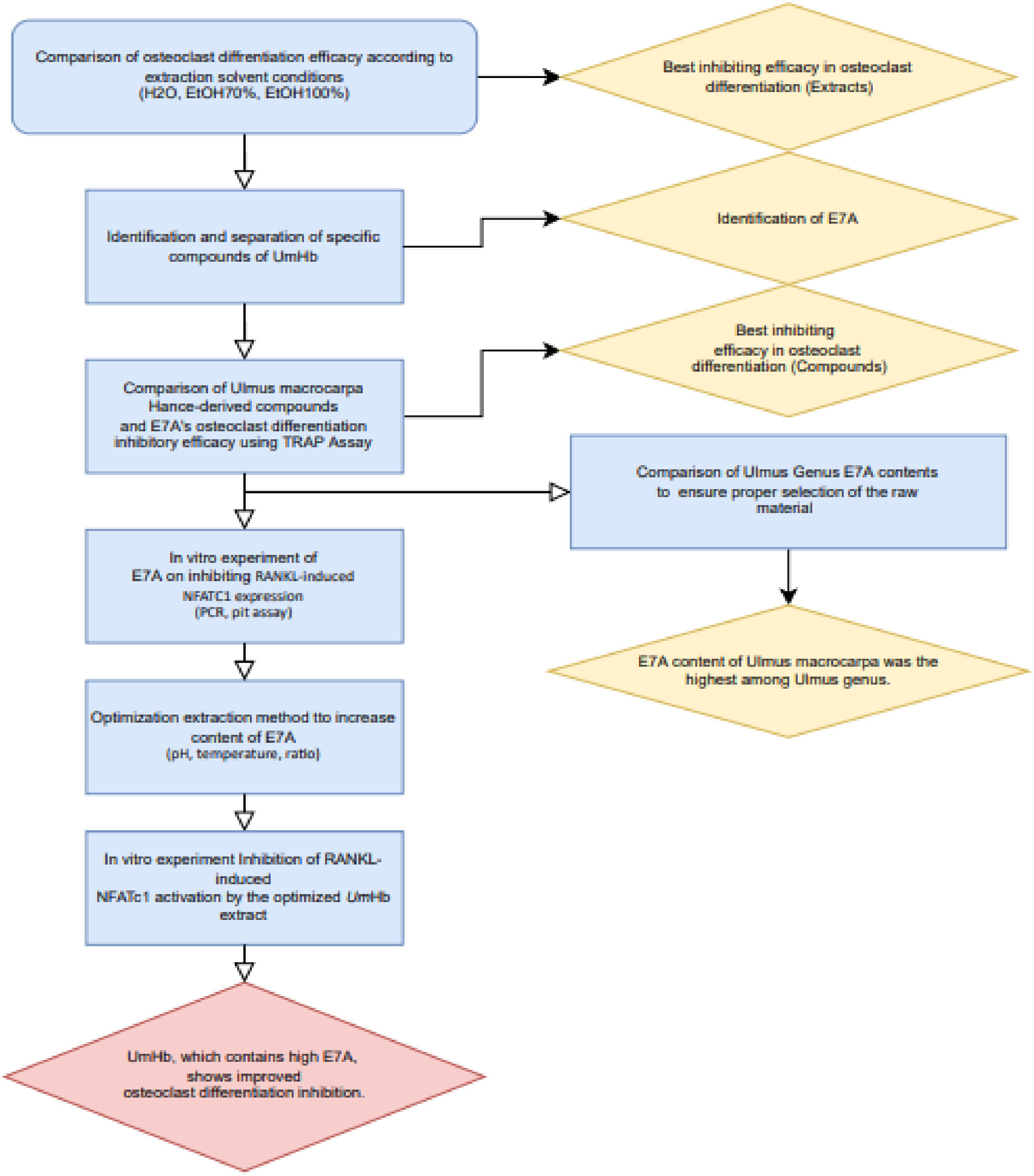

Figures S13. Uncropped western blotting images (Figure 5)

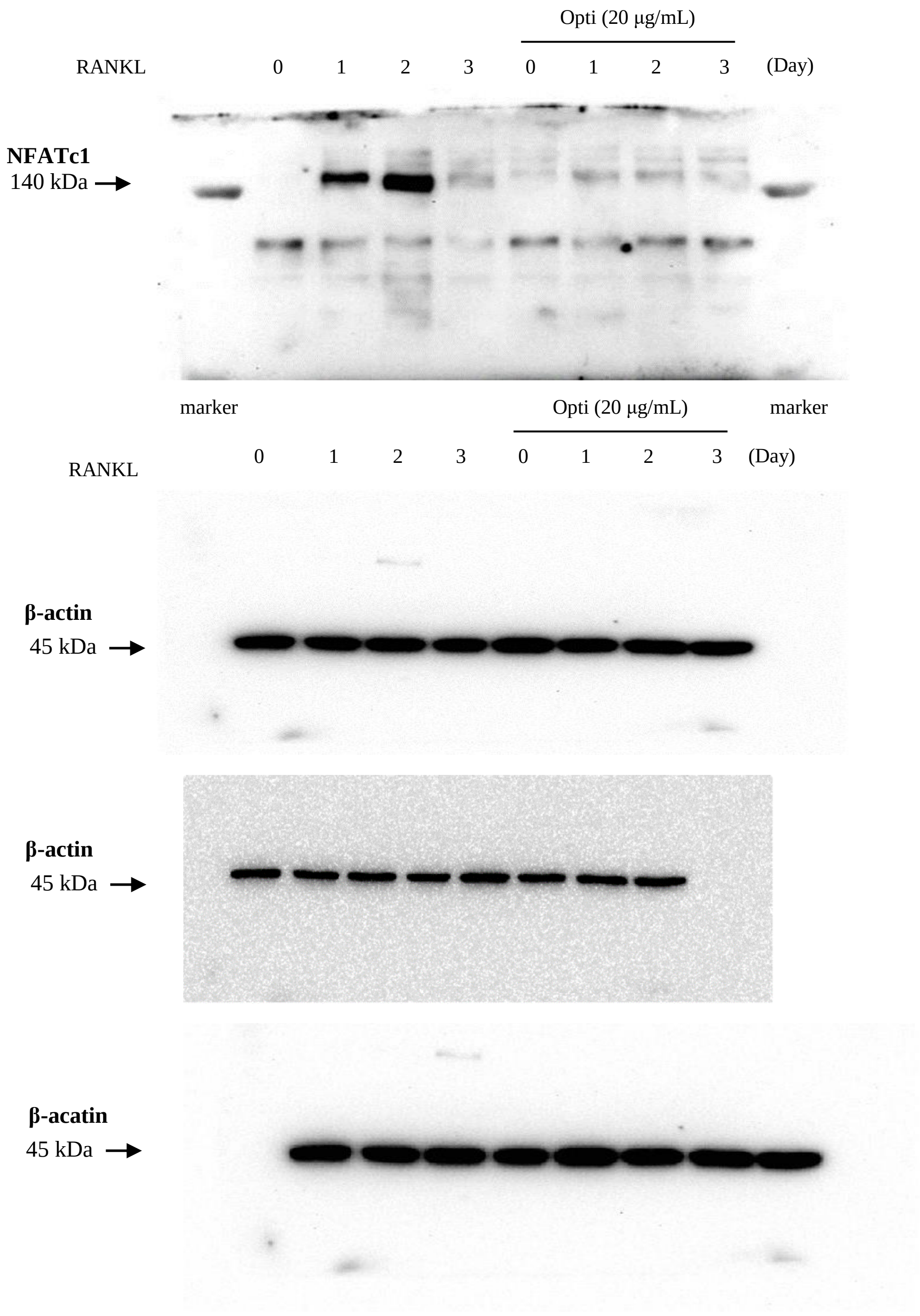

Table S1. Box-Behnken Experimental Design and Response Parameters for Obtaining E7A-containing Extracts from *Ulmus macrocarpa* Hance bark.

| Standard order | Ratio (mL/g) | Temp (°C) | pH | Time (min) | E7A content (mg/g extract) | |
|---|---|---|---|---|---|---|
| | | | | | Actual | Predicted |
| 1 | 60 | 50 | 9 | 90 | 2.939982 | 3.583875 |
| 2 | 100 | 50 | 9 | 90 | 2.923131 | 3.695148 |
| 3 | 60 | 90 | 9 | 90 | 2.958002 | 6.0283 |
| 4 | 100 | 90 | 9 | 90 | 8.488186 | 11.68661 |
| 5 | 80 | 70 | 5 | 60 | 7.16491 | 9.473598 |
| 6 | 80 | 70 | 13 | 60 | 0.093011 | -0.08326 |
| 7 | 80 | 70 | 5 | 120 | 5.606503 | 9.625085 |
| 8 | 80 | 70 | 13 | 120 | 0 | 1.533627 |
| 9 | 60 | 70 | 9 | 60 | 6.519959 | 7.161999 |
| 10 | 100 | 70 | 9 | 60 | 9.131182 | 9.142613 |
| 11 | 60 | 70 | 9 | 120 | 7.84878 | 7.142008 |
| 12 | 100 | 70 | 9 | 120 | 12.26836 | 10.93097 |
| 13 | 80 | 50 | 5 | 90 | 2.42369 | 2.715729 |
| 14 | 80 | 90 | 5 | 90 | 9.64775 | 11.69112 |
| 15 | 80 | 50 | 13 | 90 | 0.387739 | -2.35098 |
| 16 | 80 | 90 | 13 | 90 | 0.096894 | -0.89049 |
| 17 | 60 | 70 | 5 | 90 | 12.19584 | 7.613251 |
| 18 | 100 | 70 | 5 | 90 | 16.72217 | 12.64207 |
| 19 | 60 | 70 | 13 | 90 | 0 | 0.933125 |
| 20 | 100 | 70 | 13 | 90 | 0.238274 | 1.673885 |
| 21 | 80 | 50 | 9 | 60 | 1.520334 | 2.216257 |
| 22 | 80 | 90 | 9 | 60 | 11.7217 | 8.239884 |
| 23 | 80 | 50 | 9 | 120 | 3.571285 | 3.906127 |
| 24 | 80 | 90 | 9 | 120 | 12.16128 | 8.318384 |
| 25 | 80 | 70 | 9 | 90 | 6.210983 | 8.644835 |
| 26 | 80 | 70 | 9 | 90 | 6.399325 | 8.644835 |
| 27 | 80 | 70 | 9 | 90 | 9.958712 | 8.644835 |
| 28 | 80 | 70 | 9 | 90 | 10.30981 | 8.644835 |
| 29 | 80 | 70 | 9 | 90 | 10.34535 | 8.644835 |
